# Advances in label-free dynamic imaging of live cells using fourier ptychographic microscopy

**DOI:** 10.52601/bpr.2025.250017

**Published:** 2026-08-31

**Authors:** Lina Shao, Houliang He, Yong Liu, Fei Sun, Yangang Pan, Dayu Li, Hongda Wang

**Affiliations:** 1 State Key Laboratory of Electroanalytical Chemistry, Changchun Institute of Applied Chemistry, Chinese Academy of Sciences, Changchun 130022, China; 2 College of Communication Engineering, Jilin University, Changchun 130012, China; 3 State Key Laboratory of Advanced Manufacturing for Optical Systems, Changchun Institute of Optics, Fine Mechanics and Physics, Chinese Academy of Sciences, Changchun 130022, China; 4 Center of Materials Science and optoelectronics Engineering, University of Chinese Academy of Sciences, Beijing 100049, China; 5 School of Applied Chemistry and Engineering, University of Science and Technology of China, Hefei 230026, China

**Keywords:** Fourier ptychography microscope, Label-free, Super-resolution, Subcellular organelles, Long-term imaging

## Abstract

High-resolution and long-term dynamic imaging are essential for visualizing the spatial distribution and interaction networks of organelles within living cells. Although traditional super-resolution fluorescence microscopy achieves impressive resolutions of 20–100 nm, it faces significant challenges, including phototoxicity, photobleaching, and limited suitability for prolonged live-cell observation. These issues have driven the development of label-free imaging technologies that aim to minimize disruption to cellular physiology while providing high-resolution, non-destructive imaging. Among label-free approaches, Quantitative Phase Imaging (QPI) has emerged as a promising alternative for live-cell research by reconstructing cellular structures based on phase changes in transmitted light. In particular, Fourier ptychographic microscopy (FPM) achieves resolutions as fine as 150 nm while maintaining a large field of view, making it highly suitable for high-resolution, label-free imaging. Since its introduction in 2013, FPM has rapidly advanced, offering computational imaging capabilities that surpass conventional resolution limits. However, current systems are constrained by slow imaging speeds due to the sequential illumination of hundreds of LEDs. Here, we review the collective advancements in FPM that have transformed its capabilities over recent years. While numerous research groups have contributed to this progress, key innovations include the development of two-dimensional super-resolution FPM techniques that overcome the diffraction limit through iterative pattern optimization. Building upon these efforts, our group has introduced three-dimensional fast high-resolution Fourier microscopy, achieving 3D dynamic imaging at sub-micron resolution through computational refocusing algorithms. Collectively, these advancements establish FPM as a groundbreaking tool for real-time, high-resolution imaging of living cells, facilitating comprehensive analysis of organelle interactions and providing valuable insights into cellular functions and disease mechanisms.

## INTRODUCTION

Organelles are the fundamental structural and functional units of cellular life, and their interactions are crucial for maintaining normal cellular functions (Cohen *et al.* 2018). Organelle interactions play a pivotal role in regulating cellular functions (Rossini *et al.* 2021). Through these interactions, organelles collaboratively carry out a series of complex biological processes, such as material transport, energy metabolism, and signal transduction. Investigating organelle interactions not only helps elucidate the internal mechanisms of cells but also provides critical insights into the onset and progression of diseases. For instance, the interactions between mitochondria and the endoplasmic reticulum are essential for regulating calcium homeostasis and lipid metabolism, and their dysregulation is closely associated with various neurodegenerative diseases (Sammeta *et al.* 2023). Recent discoveries of novel organelles, such as PXo bodies (Xu *et al.* 2023) and stress granules (Shin and Brangwynne 2017), have significantly expanded our understanding of cellular structure and function. These novel organelles typically form under specific physiological or pathological conditions and participate in critical processes such as cellular stress responses and gene expression regulation. However, research on these novel organelles is still in its early stages, and their specific functions and mechanisms remain to be fully explored. The development of label-free imaging technologies offers a powerful tool for observing and studying these novel organelles, with the potential to drive rapid progress in this field.

The study of organelle interactions imposes multifaceted requirements on imaging technologies. First, a resolution on the order of hundreds of nanometers is essential for observing the fine structures and dynamic interactions of organelles. Given that most organelles are hundreds of nanometers in size, only imaging technologies with resolutions at this scale can clearly resolve organelle boundaries and contact sites. Second, the ability to perform long-term dynamic observation is crucial for studying the dynamic processes of organelle interactions. These interactions are continuously evolving, necessitating imaging technologies capable of maintaining stable imaging over extended periods to capture the complete interaction dynamics. Furthermore, whole-cell imaging capability is a critical demand for studying organelle interactions. Organelle interactions often involve the synergistic actions of multiple organelles, requiring observation and analysis in the context of the entire cell. This demands imaging technologies with large fields of view and high resolution, capable of presenting the spatial distribution and interaction networks of organelles at the single-cell level. Additionally, to minimize the interference with the normal physiological state of cells, ideal imaging technologies should exhibit low phototoxicity and eliminate the need for fluorescent labeling, enabling long-term, non-destructive observation of live cells.

Super-resolution fluorescence microscopy, as a rapidly advancing imaging technology in recent years, has demonstrated significant advantages in organelle interaction studies. Its most notable feature is that can break through the resolution limit of traditional optical microscopes, achieving resolutions of 20–100 nm (Gustafsson 2000; Hell and Wichmann 1994; Rust *et al.* 2006), thereby clearly revealing the fine structures of most organelles. Moreover, fluorescence labeling technology provides high contrast, allowing specific organelles or molecules to be accurately identified and localized within complex cellular environments. These advantages make super-resolution fluorescence microscopy an important tool for studying organelle structures and interactions, particularly in elucidating spatial relationships and dynamic processes between organelles. However, its application in live-cell imaging also faces several limitations (Dong *et al.* 2020; Hoebe *et al.* 2007; Ma *et al.* 2019). First, high-intensity laser illumination may induce phototoxicity, thereby affecting the normal physiological functions of cells. Second, fluorescent molecules are prone to photobleaching under prolonged illumination, limiting long-term observation of organelle dynamics. Additionally, the fluorescence labeling process itself may perturb the normal cellular state, and currently, it is not feasible to simultaneously label all organelles or molecules of interest. These limitations have prompted researchers to continuously explore label-free imaging technologies more suitable for long-term live-cell observation, aiming to reduce interference with cellular physiology and achieve non-destructive monitoring of organelle interaction processes. These challenges have fueled the demand for novel imaging technologies, particularly label-free imaging methods.

Quantitative Phase Imaging (QPI) technology, as a label-free imaging approach, has shown significant advantages in live-cell research (Nguyen *et al.* 2022). This technology reconstructs cellular structures by detecting phase changes in light waves passing through the sample, obtaining high-contrast cell images without fluorescent labeling, thereby effectively avoiding phototoxicity and photobleaching issues associated with fluorescence labeling. This characteristic makes it particularly suitable for long-term, non-destructive dynamic observation of live cells. Additionally, QPI can provide quantitative biophysical information, such as cell dry mass, offering a new measurement dimension for studying organelle dynamics. Currently, QPI technologies mainly include Digital Holographic Microscopy (DHM), Differential Phase Contrast Microscopy (DPC), Optical Coherence Tomography (OCT), and Fourier Ptychographic Microscopy (FPM). These methods rely on the measurement and reconstruction of light wave phase information, providing high-contrast, quantitative cell imaging in a label-free, low-phototoxicity manner. Among them, DHM employs interferometric measurement techniques to achieve high-precision phase imaging of cells and subcellular structures by recording and reconstructing digital holograms, with resolutions reaching the submicron level (Cuche *et al.* 1999). DPC enhances phase gradient signals through modulated illumination modes, improving imaging capabilities for transparent samples, with typical resolutions of 200–300 nm (Tian and Waller 2015). OCT, based on low-coherence interferometry, can obtain deep structural information from biological tissues, with resolutions typically ranging from 1 to 10 μm, suitable for *in vivo* tissue imaging (Fujimoto 2003). FPM combines phase retrieval algorithms and multi-angle illumination strategies to break through the resolution limits of traditional optical microscopes via computational imaging, achieving resolutions as high as 150 nm while also offering the advantages of a large field of view (Shao *et al.* 2024).

Fourier ptychography (FP), which was first invented by Guoan Zheng *et al.* in 2013 (Zheng *et al.* 2013), offers an alternative way to develop a high-resolution label-free technique. A typical FP microscopy (FPM) setup consists of a light-emitting diode (LED) array mounted under the sample and a light microscope with a low-NA objective lens (Zheng 2014). The FPM iteratively stitches together a series of variably illuminated, low-resolution intensity images by the LED array in Fourier space to produce a complex, wide-field, high-resolution sample image. Jiasong Sun *et al.* reported a resolution-enhanced FPM (REFPM) platform and used an oil-immersion condenser to achieve a synthetic numerical aperture of 1.6 with a 10×/0.4 NA objective using a dense LED board, attaining a lateral resolution of around 150 nm with an incident wavelength of 435 nm (Sun *et al.* 2017). Pan *et al.* developed a super-resolution FPM (SRFPM) platform and enabled the ultimate performance of 1.05 synthetic NA at a lateral resolution of 244 nm with an incident wavelength of 465 nm via a 4×/0.1 NA objective (Pan *et al.* 2018). These two systems, which both achieve an ultra-high resolution under a low-power objective lens, have the best imaging performance at present. However, the REFPM and SRFPM platforms use 261 and 415 LEDs, respectively, and all the LEDs are lit up one by one, resulting in slow imaging acquisition speed, which cannot meet the requirements of real-time dynamic imaging.

In recent years, our research group, in collaboration with the team of Dayu Li, has made significant strides in advancing Fourier Ptychographic Microscopy (FPM), contributing to multiple key innovations in the field. This paper presents a comprehensive overview of these breakthroughs, focusing on several critical aspects that have significantly enhanced the capabilities and application of FPM in biological imaging. First, we introduce the development of two-dimensional super-resolution Fourier ptychographic microscopy, which breaks the resolution limitations of traditional microscopy, offering higher spatial resolution and the ability to capture more detailed information (Shao *et al.* 2024). Next, we focus on the development of three-dimensional fast high-resolution Fourier microscopy, which, through advanced optical design and imaging techniques, achieves fast, high-resolution imaging in three-dimensional space, significantly improving the imaging efficiency and accuracy of biological samples (Sun *et al.* 2022). In addition, the research group has conducted in-depth studies on reconstruction algorithms and proposed new optimization algorithms to enhance the accuracy and speed of image reconstruction, providing more reliable technical support for practical applications. Moreover, significant progress has been made in the study of wavevector correction, addressing imaging errors caused by wavevector information deviation in traditional Fourier microscopes, thereby further improving imaging quality (He *et al.* 2025).

## PRINCIPLE OF FPM

### Hardware system

**Figure 1:**
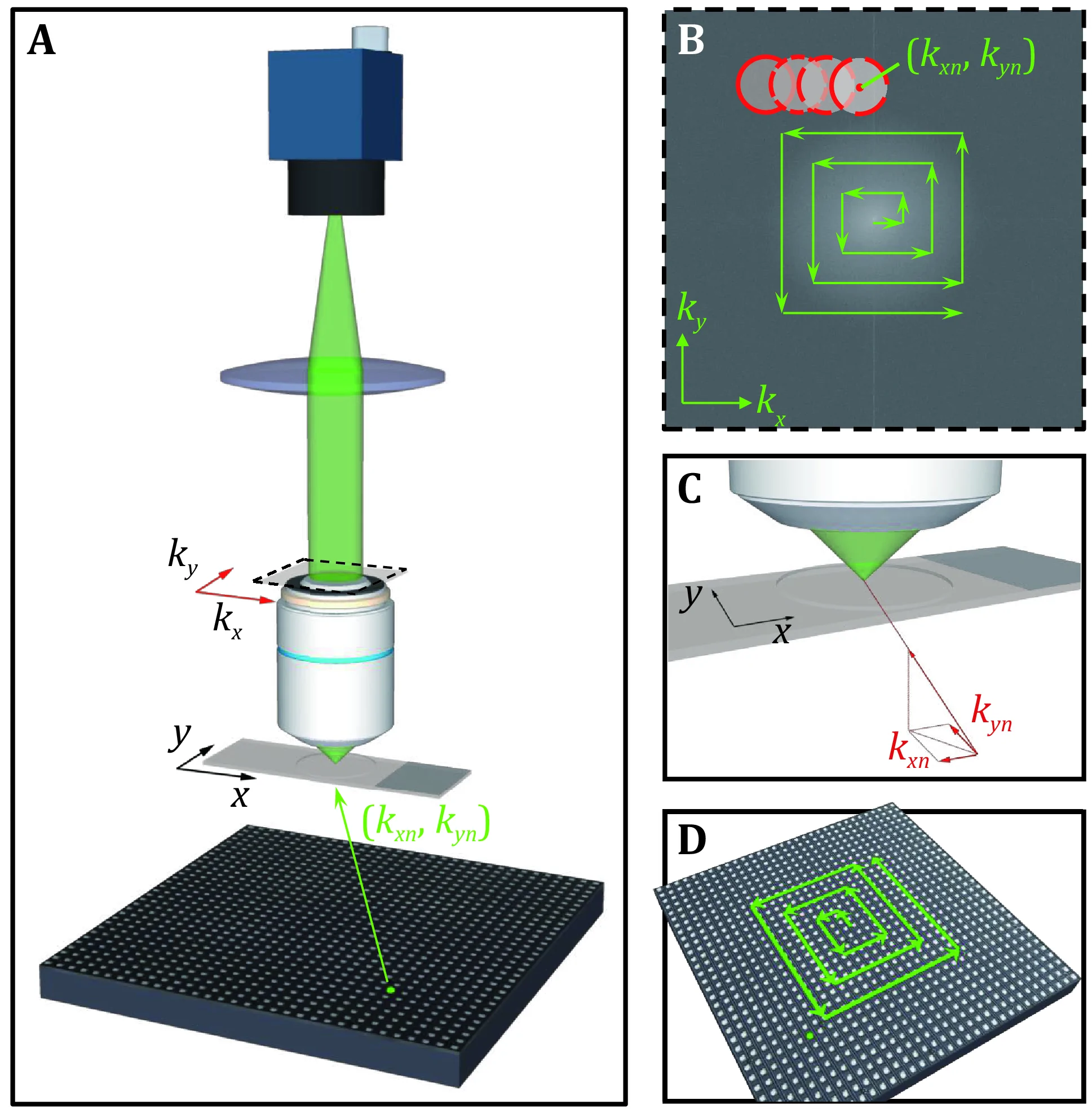
Optical imaging principle and system setup of FPM. **A** System setup of FPM. A programmable LED matrix is placed beneath the sample and sequentially illuminated, one LED at a time. The *n*th LED illuminates the sample with a wave-vector (*k*_*xn*_, *k*_*y*n_). **B** The object’s finite spatial frequency support, determined by the microscope's numerical aperture (NA) in the Fourier domain (red circle), is shifted to reflect each unique LED illumination angle. The Fourier transforms of multiple shifted low-resolution measurements (each represented by a circle) are combined to extend the resolution of the complex sample spectrum beyond the objective lens’s cutoff. **C** Light emitted from a single LED illuminates a small sample area with a wave-vector (*k*_*xn*_, *k*_*y*n_). **D** LEDs are sequentially activated during FPM image acquisition

Fourier ptychographic microscopy (FPM) is a large-field, high-resolution quantitative phase contrast imaging technique proposed by Zheng *et al.* in 2013 (Zheng *et al.* 2013). This method integrates the concepts of phase recovery and synthetic aperture. A traditional Fourier ptychographic imaging system is shown in Fig. 1. In this system, a programmable LED array replaces the light source and condenser used in traditional optical microscopes. By sequentially illuminating different LED units at different positions, the system provides plane-wave illumination from various directions, while simultaneously capturing a series of low-resolution raw images (Ou *et al.* 2013). Each low-resolution image corresponds to a different sub-frequency region of the sample's spectrum. The center of these circular sub-frequency regions is determined by the illumination wavevector, while the radius of the region is jointly determined by the objective lens's numerical aperture and the illumination wavelength.

### Algorithmic principle

**Figure 2:**
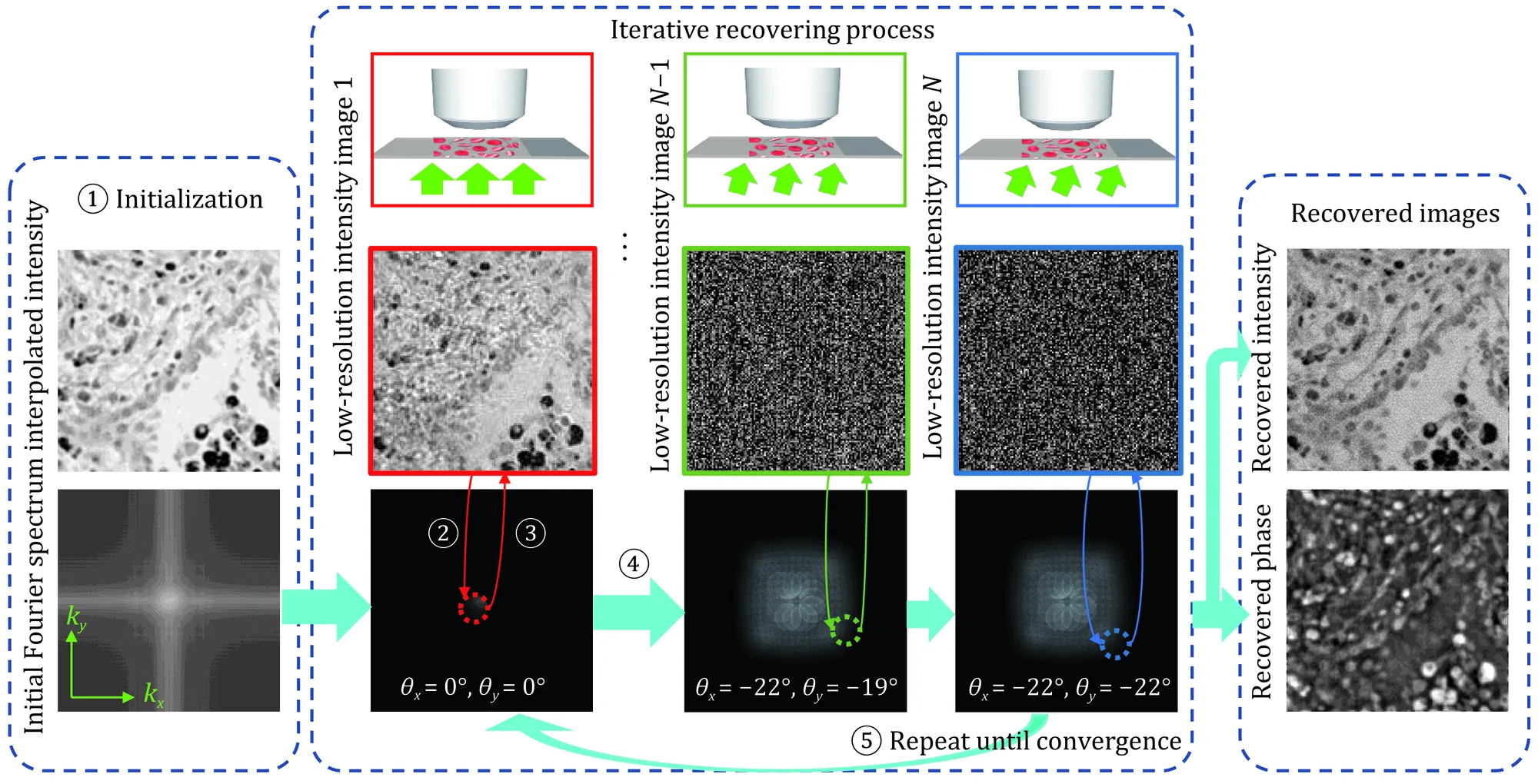
Flow diagram of iterative reconstruction of FPM

The reconstruction process of Fourier ptychographic imaging involves finding the high-resolution complex image solution of the object, constrained by the captured low-resolution images (Fig.2). This process can be viewed as an optimization problem, where the goal is to minimize the difference between the low-resolution images generated by the reconstructed complex image of the object during the iterative process and the captured low-resolution images. Traditional Fourier ptychographic imaging algorithms alternate between the spatial and frequency domains to reconstruct the high-resolution complex image information of the object. During the reconstruction, two types of constraints are utilized:

(1) In the spatial domain, the captured low-resolution images are used as the amplitude constraint for the optimal solution.

(2) In the frequency domain, the optical transfer function of the objective lens (a circular aperture function) is used as the frequency support constraint for the optimal solution, corresponding to different illumination angles. This circular aperture scans and stacks in the frequency spectrum to form a larger frequency bandwidth, which helps recover the high-frequency information of the object.

## RESEARCH PROGRESS

### Super-resolution imaging technology

**Figure 3:**
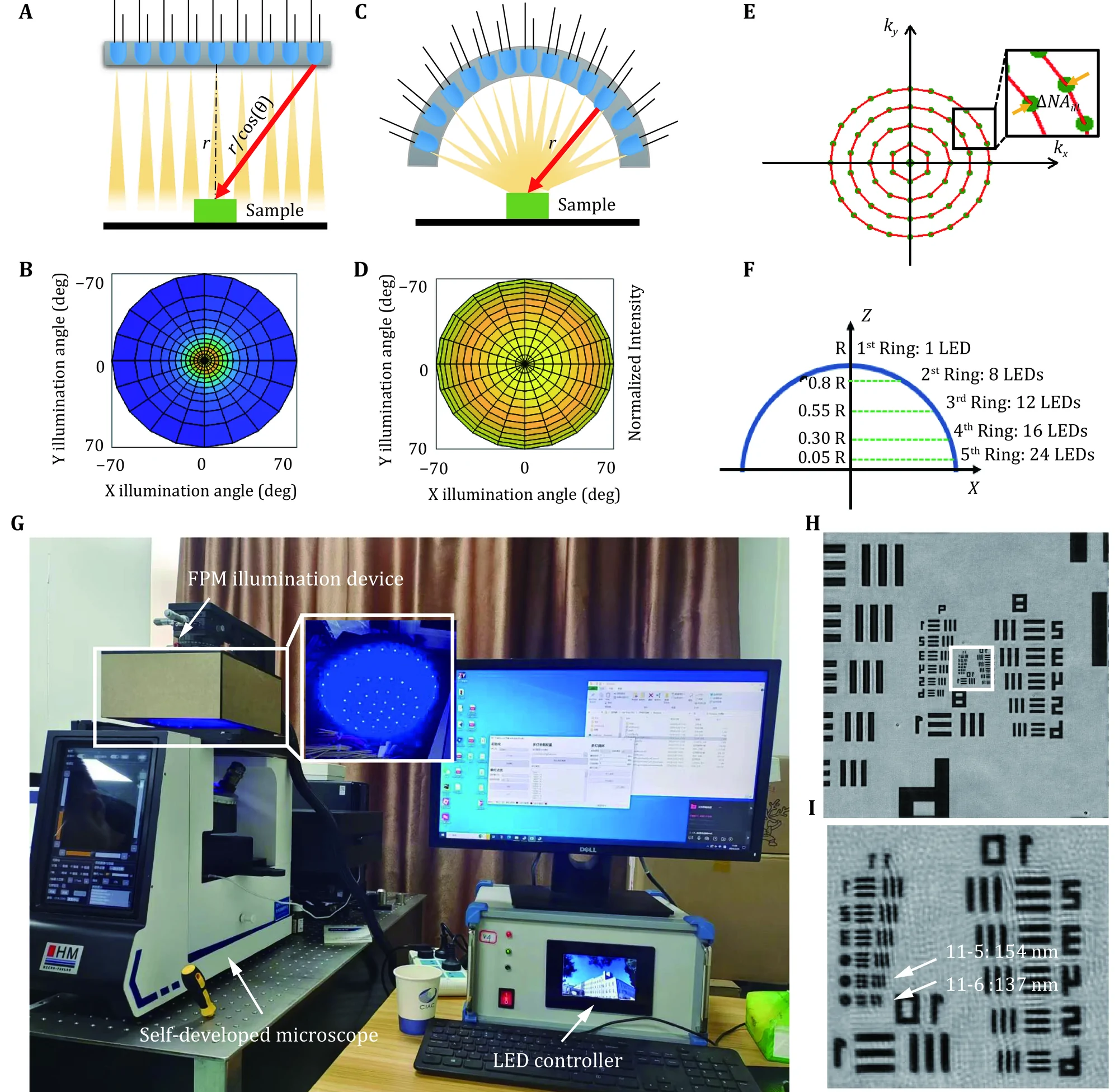
**A**,**B** Schematic comparison of planar and domed LED arrays. **C**,**D** Relative mean pixel intensities of constructed planar vs domed arrays as a function of LED angle. **E**,**F** The arrangement of spectral concentric circles. **G** Experiment system and the structure diagram of the hemispherical light source. **H**,**I** Reconstructed results of USAF target with 40×/0.6NA objective

In traditional FPM systems, flat LED arrays are commonly used as illumination sources. However, due to the emission characteristics of LEDs, when the illumination angle is large, the light intensity received by the sample significantly attenuates, directly affecting the signal-to-noise ratio of image acquisition and consequently reducing reconstruction resolution and image quality (Figs. 3A and 3C). Although increasing exposure time can partially mitigate this issue, it significantly prolongs acquisition time, thereby reducing the overall acquisition efficiency of the FPM system. Additionally, the structural design of flat LED arrays presents further challenges. The spectrum overlap rate of edge LEDs is much higher than that of central LEDs, leading to increased information redundancy. Under the same illumination numerical aperture (NA) conditions, the system requires more LEDs to cover the required spectrum range, which not only increases system complexity but also further limits the acquisition efficiency of the FPM system.

To address this issue, inspired by the work of Pan *et al*. (Pan *et al.* 2018), this study adopts a hemispherical illumination structure to mitigate the brightness attenuation caused by flat-panel LEDs. On one hand, this structure compensates for the intensity loss due to the LED's Lambertian emission, and on the other hand, it ensures that the distance between the sample and each LED is consistent. In this configuration, the light intensity attenuation improves from cos^4^(*θ*) for a flat-panel LED array to cos(*θ*) (Figs. 3A−3D). The hemispherical structure uses a hexagonal arrangement, where the spacing between adjacent points is uniform. Let the number of rings be *m* = 0, 1, 2, 3, …, then for each ring, except for the center point, the number of LEDs is 6*m*. LEDs in each ring are evenly distributed along the circumference, with a consistent radial wavevector spacing Δ*NA*_ill_ between adjacent rings. From the geometric relationship, it can be seen that the illumination wavevectors of any two adjacent LEDs in this structure are approximately Δ*NA*_ill_, ensuring uniform overlap across the entire frequency spectrum (Figs. 3E and 3F).

Finally, we built an FPM system based on 61 LED light sources on our self-developed inverted microscope (Fig. 3G), achieving a numerical aperture (NA) of 0.98. When combined with a 40×/0.6 NA objective lens, this system breaks the optical diffraction limit, reaching a lateral resolution of 150 nm (Fig. 3H), while also offering high-speed imaging capability of >1 Hz. It covers a field of view of 118 μm × 118 μm and can support continuous monitoring for up to 4 h. With a 100×/1.45 NA objective lens, the resolution reaches approximately 110 nm (supplementary Figs. S1 and S2). This system enables label-free observation of biological processes such as vesicle transport, mitochondrial fusion and fission, and cell fusion, marking a significant breakthrough in live-cell imaging technology. It provides new analytical tools for cell biology research.

### Image reconstruction algorithms

In the FPM systems, the problem to solve is to recover the high-resolution image (including amplitude and phase) of the sample from a series of low-resolution intensity measurements taken at different illumination angles. To reconstruct the high-resolution image of the sample, we can construct a loss function based on the captured actual low-resolution intensity images, and use an iterative optimization algorithm to minimize the loss function, progressively converging to the minimum value. The reconstruction of the high-resolution complex image of the entire sample can thus be viewed as a nonlinear minimization problem based on the given loss function. Gradient descent can be used to minimize the loss function, thereby optimizing the solution for the high-resolution image in Fourier ptychographic imaging (Sun *et al.* 2019, 2021).

In traditional Fourier ptychographic imaging, during the iterative calculations, the signal intensities of the bright-field and dark-field images differ by two orders of magnitude, with the dark-field image having a much lower signal-to-noise ratio than the bright-field. In the overlapping region of the bright-field and dark-field, the update of the dark-field image can actually degrade the reconstruction quality of the high-resolution image. Therefore, it is necessary to explore methods where only the bright-field image can be updated in the overlapping region. Additionally, the update sequence for each sub-aperture in the frequency domain is usually based on the numerical aperture of the LED illumination, from low to high. Experiments have shown that in the overlapping region of two adjacent sub-apertures, the sub-aperture updated first has a greater impact on the final result, causing an uneven update weight across different regions of the frequency domain. To address this issue, we need to explore methods that allow simultaneous updates of the sub-apertures, ensuring more balanced update weights for each sub-aperture.

**Figure 4:**
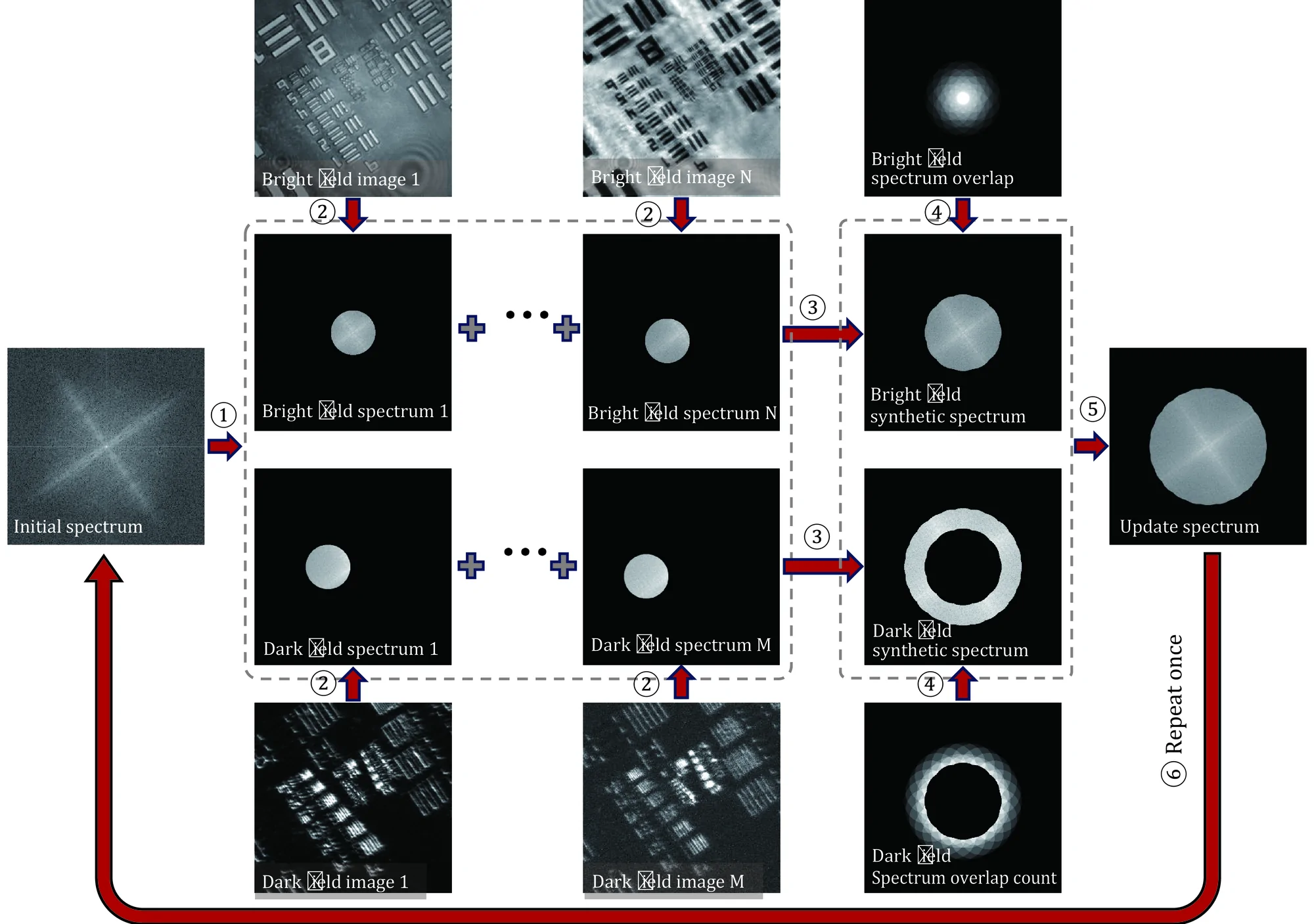
Iterative reconstruction procedure of FPM

We proposed a synchronous update algorithm for both bright-field and dark-field in FPM reconstruction, as shown in Fig. 4. First, we initialize a high-resolution complex image, perform a Fourier transform on it to obtain the frequency spectrum in the Fourier domain, and decompose this spectrum into a series of sub-aperture regions corresponding to different illumination wavevectors. Based on the illumination wavevectors, the sub-aperture regions are divided into bright-field and dark-field spectra. Next, using gradient descent, we update the spectrum of each sub-aperture region. Third, the sub-region spectra of the bright-field and dark-field are added together separately to obtain the synthesized bright-field and dark-field spectra. Fourth, we establish bright-field and dark-field overlap count maps to correct for the overlapping interference in the synthesized bright-field and dark-field spectra. Fifth, the corrected bright-field and dark-field synthesized spectra are combined to update the high-resolution spectrum. Finally, the updated high-resolution spectrum replaces the initial spectrum, and the above steps are repeated through multiple iterations to obtain the high-resolution complex image spectrum. The high-resolution amplitude and phase images of the sample are then obtained by performing an inverse Fourier transform on this spectrum to convert it into the spatial domain.

This method effectively eliminates dark-field noise in the overlapping region of the bright-field and dark-field by updating their respective spectra separately. Simultaneously updating the sub-aperture spectra in both bright-field and dark-field ensures more balanced update weights for each sub-aperture. Moreover, updating the sub-apertures simultaneously is advantageous for parallel computation, thereby reducing reconstruction time.

### Three-dimensional imaging

In the biomedical field, achieving high-resolution three-dimensional imaging of thick samples is of pivotal importance. Currently, commonly used three-dimensional imaging technologies, including laser confocal microscopy, two-photon microscopy, and light sheet microscopy, each have their own advantages and limitations. Laser confocal microscopy excels in three-dimensional high-resolution imaging, particularly for thin samples. However, its phototoxicity is significant, and its depth imaging capability for thick samples is limited. Two-photon microscopy offers significant advantages in depth imaging, reducing photodamage, and is more suitable for dynamic observation of living tissues; however, its imaging speed is slow, and it relies on fluorescent labeling. Light sheet microscopy is known for its fast three-dimensional imaging and strong penetration, suitable for observing thick samples, and can effectively reduce photodamage. However, its resolution is relatively low.

Since its introduction, the FPM system has been widely used for two-dimensional reconstruction of samples. However, by combining illumination from different angles with image inversion, it is also possible to deduce the three-dimensional refractive index information of the sample through a reasonable imaging model. Using LEDs as an incoherent light source, FPM can achieve three-dimensional reconstruction of unlabeled samples. However, the main challenges at present are the limitations of illumination angles and spectrum coverage, which lead to reconstruction artifacts and reduce the accuracy of three-dimensional imaging. Additionally, volumetric reconstruction requires more complex imaging models and algorithms (such as multi-layer diffraction models based on the Rytov approximation), which results in high computational costs and long processing times.

**Figure 5:**
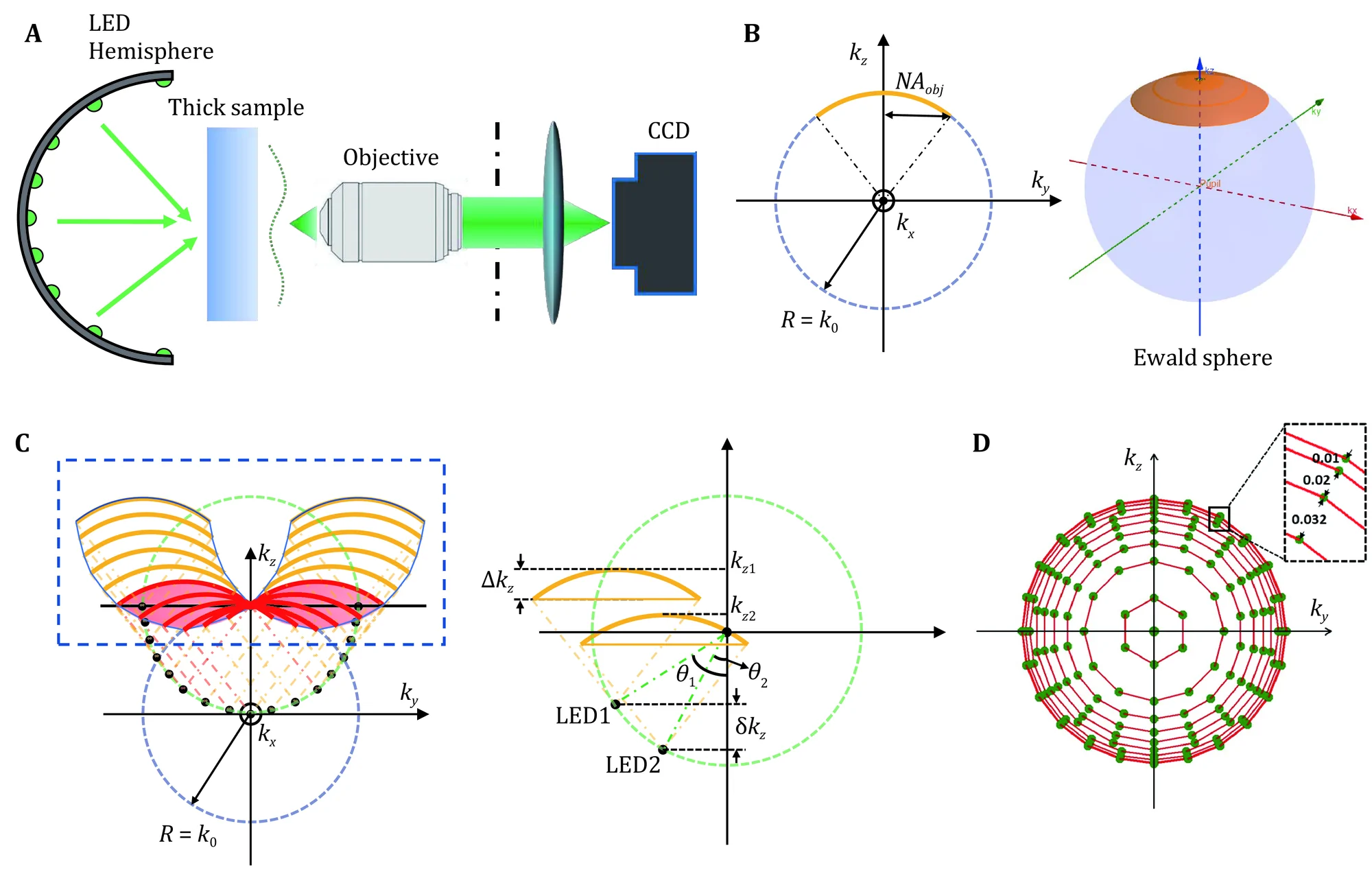
**A** Schematic of the FPM imaging system. **B** Movement of various LED illumination wave vectors on the Ewald sphere surface. **C** Ewald sphere surface and the 3D pupil function of the system. **D** Arrangement of illumination NA of the designed source

In this study, we utilize the concept of the Ewald sphere to convert light propagation and scattering information into spectrum data, thereby reconstructing the three-dimensional structure of the sample. Due to the limitations of the optical system’s objective lens and detector, the objective lens can only detect information from a certain portion of the Ewald sphere’s shell (Figs. 5A and 5B). By designing a specific LED array layout, we can access different regions of the Ewald sphere's shells, thus acquiring more scattering wavevector information (Fig. 5C). This multi-angle illumination enhances the richness of the spectrum data and provides sufficient support for three-dimensional reconstruction.

**Figure 6:**
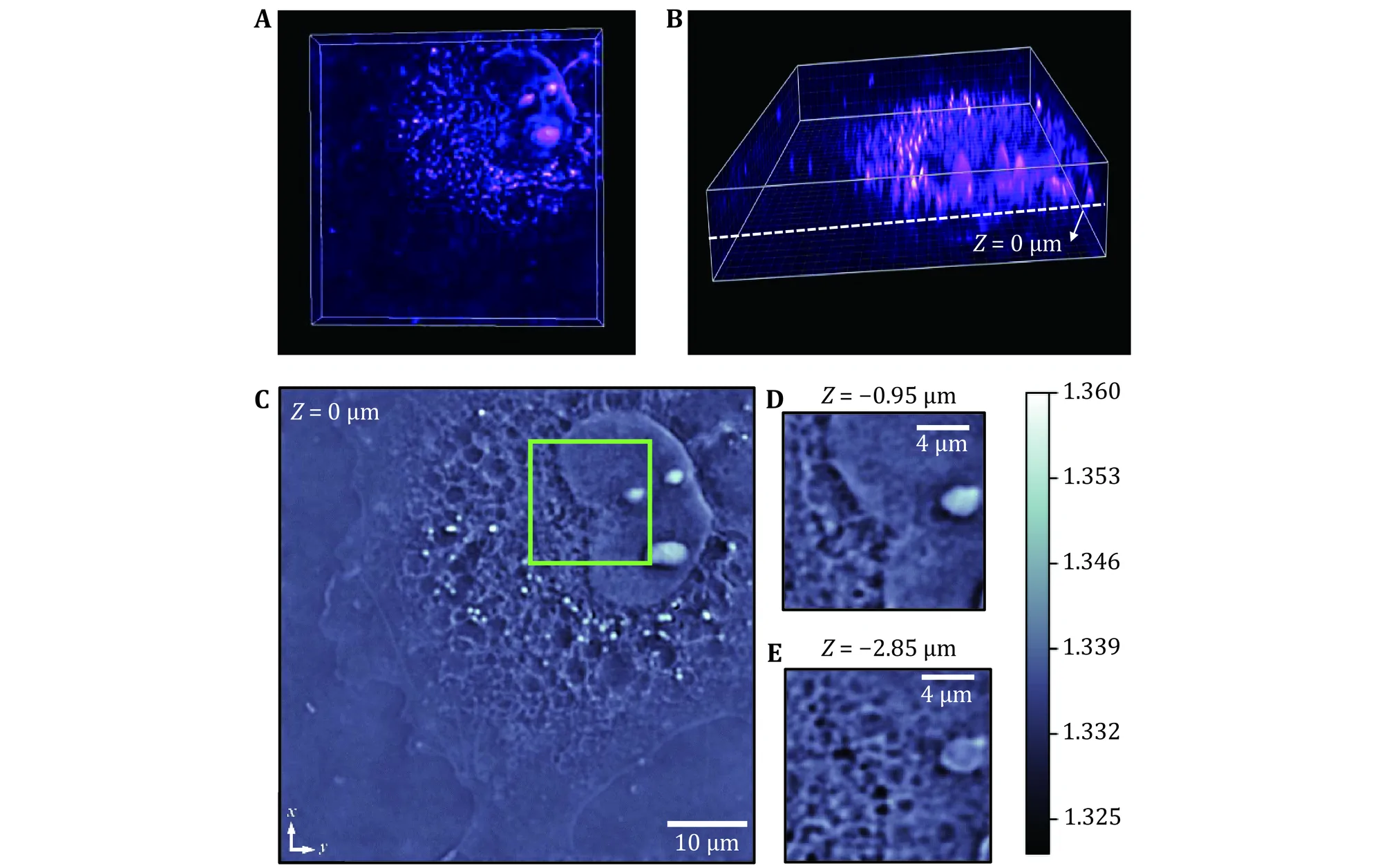
Reconstructed 3D RI of a COS-7 cell under single LED sequential illumination with 40×/0.6NA objective. **A**,**B** 3D rendering of the recovered result. **C** *X*-*Y* plane of the reconstructed 3D RI at *z* = 0 µm. **D**,**E** Enlarged area within the green box in (**D**) at different depths

Ultimately, this study redesigned the LED array structure, developing a hemispherical illumination source with 187 LEDs suitable for three-dimensional reconstruction (Fig. 5D), achieving an illumination numerical aperture (NA) of 0.98, which theoretically results in lateral and axial resolutions of approximately 150 and 500 nm, respectively (Sun *et al.* 2022). Compared to the work of Chao Zuo *et al.* (Zuo *et al*. 2020), the number of LEDs required is significantly reduced. In addition, the study proposes an illumination method based on multi-LED multiplexed coding, which greatly reduces the number of images required, thereby improving the system's acquisition frame rate from 0.53 Hz with single-point illumination to 1.27 Hz, enabling three-dimensional dynamic imaging of live cells (Fig. 6). This improvement significantly enhances the application potential of FPM technology in dynamic high-resolution three-dimensional imaging of biological samples.

### Wave vector correction

In practical engineering applications, FPM may suffer from minor positional deviations in the LED array assembly, which could lead to artifacts in the reconstructed image and a reduction in resolution (Zhu *et al.* 2022). To address this issue, we proposed a novel wave vector calibration method based on imaging circle offset and diffraction analysis, overcoming the limitations of traditional calibration techniques and providing a new solution for high-precision microscopic imaging.

**Figure 7:**
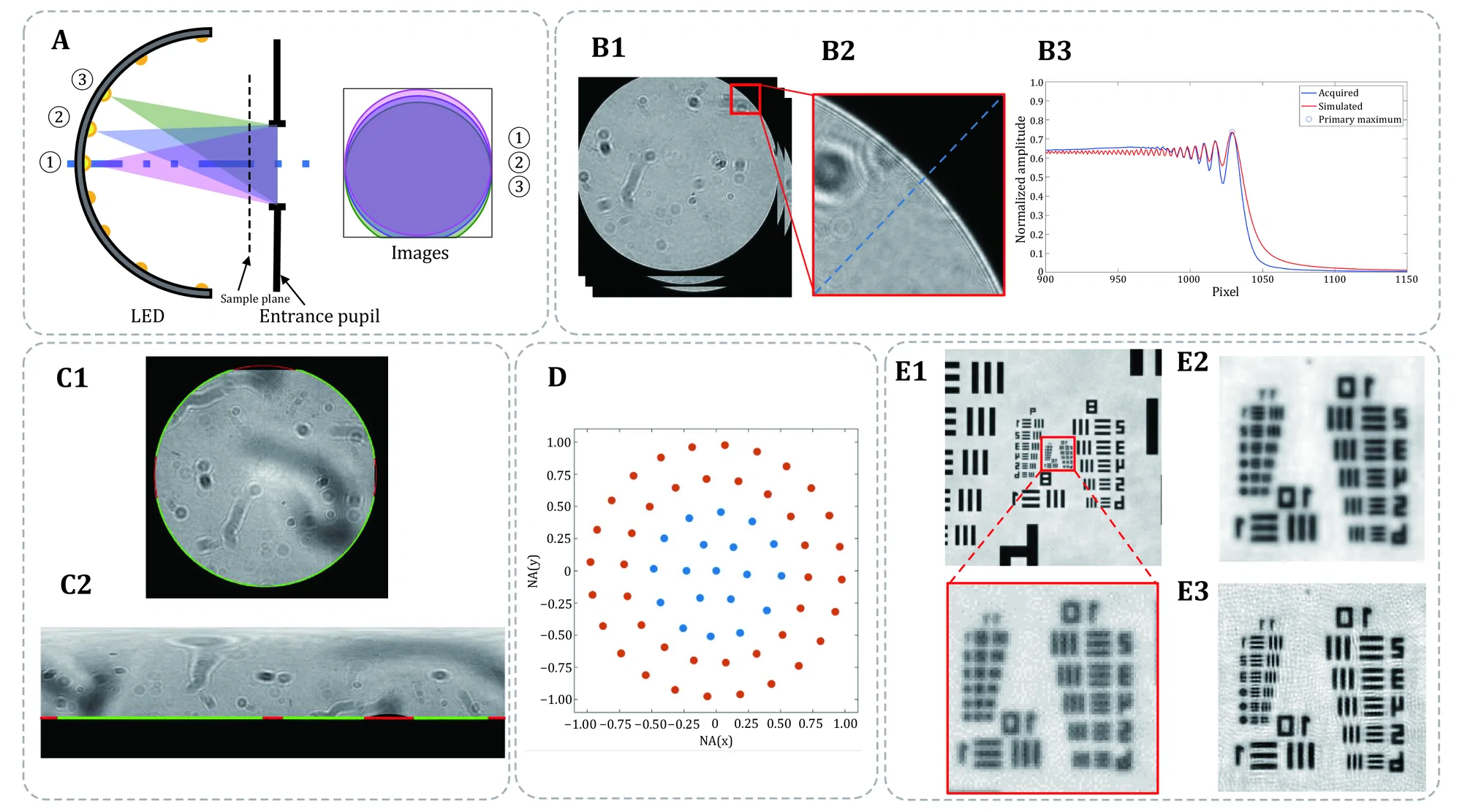
**A** Conjugate optical model of 'sample plane-entrance pupil-LED'. **B** Circular bright area in bright field image (**B1**), local enlargement of the bright area edge (**B2**), and comparison between the grayscale distribution curve along the radial direction in bright field image (blue) and simulation curve (red) (**B3**). **C** Boundary selection diagram along the peak of the first diffraction ring (**C1**), and unwrapped image after polar coordinate transformation (**C2**). **D** Corrected incident wave vector, blue correspond to bright field, brown to dark field. **E** Low-resolution image (**E1**), along with reconstruction results without wave vector correction (**E2**), and with wave vector correction (**E3**)

In an FPM system, planar displacement and rotational deviations of the LED light source significantly affect the position and size of the imaging circle (the circular bright area in the bright-field image). By incorporating a matching lens with 0.7× magnification, we clearly captured the dynamic offset of the imaging circle on the camera target plane during the switching of bright-field LEDs (Fig. 7B1). Based on this, we established a “sample plane-pupil-LED” conjugate optical model (Fig. 7A), revealing the quantitative relationship between the radius of the imaging circle and the height of the LED. However, the radius difference of the imaging circles between LEDs at different heights is extremely small (indistinguishable to the naked eye), and the boundary exhibits a gray-scale gradient zone of over 50 pixels (Fig. 7B2), which makes the traditional circular fitting method prone to significant errors. To address this issue, we turned to diffraction effect analysis: under high-magnification objectives (*e*.*g*., 40×), the interior of the imaging circle presents regular radial diffraction rings (Fig. 7B2), whose distribution is governed by the Fresnel-Kirchhoff diffraction theorem. By extracting the peak of the first diffraction ring as the boundary marker (Fig. 7C1), and combining polar coordinate transformation with circular fitting (Fig. 7C2), we achieved high-precision measurement of the pupil parameters, with the simulated diffraction curve closely matching the experimental diffraction curve (10 wave peaks aligned, Fig. 6B3). For dark-field LEDs where diffraction rings cannot be directly observed, we utilized the system translation/rotation parameters obtained from bright-field LED fitting, combined with the prior relationships from the light source design, to deduce their positions, ultimately generating corrected incident wavevectors (Fig. 7D). Experimental results showed that this method achieves calibration errors of less than 1 μm and 0.02° within a translation range of ±2 mm and a rotation range of ±5°.

After wave vector calibration, the sharpness of the line-edge contrast was significantly improved, and the lateral resolution reached an ideal 150 nm (Figs. 7E1−7E3). This study represents the first instance of integrating the pupil cutoff effect with diffraction analysis, successfully overcoming the long-standing challenge of LED position calibration and laying a solid foundation for the development of a robust FPM system.

## APPLICATIONS

### Cell panorama imaging reveals a novel cellular structure

Vesicles and other membrane transport carriers constitute the intracellular logistics system and play an important physiological role. However, research on the types and functions of intracellular vesicles remains insufficient, and new technologies are needed to provide further insights. In 2021, Liangyi Chen's group at Peking University discovered a new subcellular structure called black vesicles using ODT microscopy for live-cell imaging (Dong *et al.* 2020). However, the composition and function of these black vesicles, as well as the potential cooperation with other organelles, are still unclear. We developed a super-resolution, label-free FPM and combined it with fluorescence confocal techniques to conduct research on the black vesicles. It further demonstrates the morphology, distribution, movement, and effect of the cell cycle on the black vesicles, laying the foundation for confirming whether black vesicles are a novel organelle.

**Figure 8:**
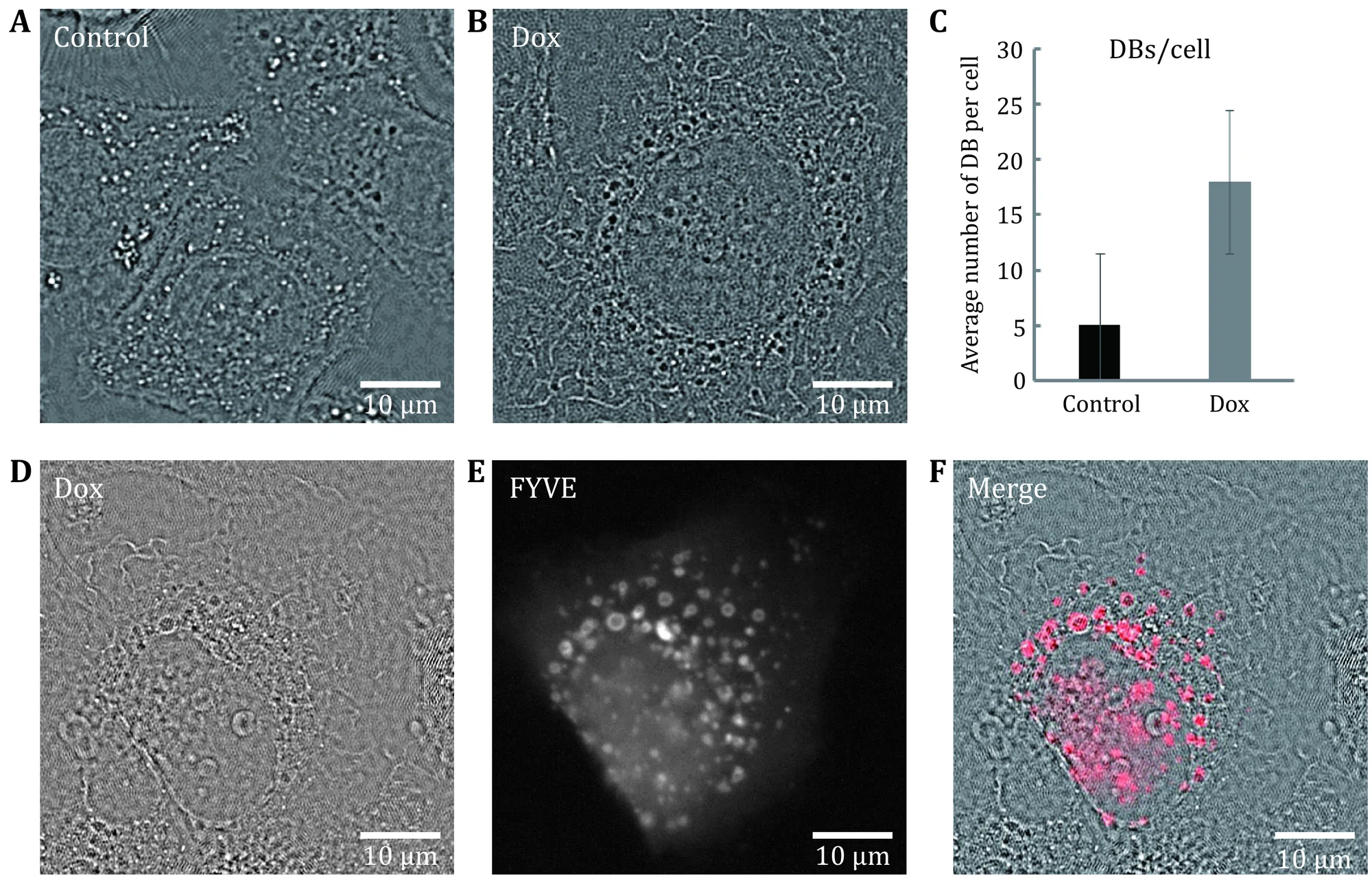
Dox promoting senescence in A549 cells and colocalization of Dox with FYVE. **A** Normal cultured A549 cells. **B** A549 cells with added Dox stimulation. **C** comparison of the number of DBs in normal cultured A549 cells between aged A549 cells. **D** A549 cells transfected with FYVE-EGFP after Dox treatment. **E** FYVE-EGFP fluorescence of A549 cells. **F** FYVE-EGFP fluorescence and DBs colocalization image

In our study (Liu *et al.* 2024), we primarily analyzed the black vesicles by stimulating lipid droplets and endosomes, promoting cellular endocytosis, and inducing cell aging. Eventually, we found that black vesicles have a weak correlation with lipid droplets and endocytic vesicles, whereas they exhibit a significant correlation with late endosomes (Fig. 8). Moreover, both the number and size of black vesicles increase significantly during cell aging.

This research highlights the unique value of FPM technology in investigating organelle interactions. Its label-free feature not only circumvents the phototoxicity constraints associated with fluorescent probes but also enables the capture of the spatiotemporal dynamics of multiple organelles through panoramic phase imaging. This breakthrough paves the way for exploring the energy metabolism characteristics of black vesicles and their cooperative mechanisms within the lysosome-endosome network. In the future, we plan to develop an integrated system combining FPM and Raman spectroscopy, which will facilitate the cross-scale correlation analysis of chemical composition and mechanical properties of biological samples under the label-free framework.

### Long-term live cell imaging reveals cell-cell fusion

We conducted dynamic imaging observations of the cell−cell fusion process for over four hours using our self-development FPM system. Virus-induced cell−cell fusion plays an important role in viral replication and in evading the host immune system’s attacks (Plescia *et al.* 2021). Since cell−cell fusion shares similarities with virus−cell fusion in terms of membrane fusion mechanisms, studying the mechanism of cell−cell fusion provides important clues for identifying potential drugs to inhibit viral invasion.

Traditional live-cell dynamic experiments often rely on confocal microscopy, but confocal microscopy has limitations in terms of field of view and dependence on fluorescent markers for target observation. Moreover, live-cell fluorescence imaging faces phototoxicity issues in drug screening, which restricts the duration of imaging. In contrast, FPM technology, as a label-free imaging technique, offers advantages such as high throughput, low phototoxicity, and long-duration imaging. It can capture the dynamic changes of the cell fusion process over extended periods without relying on fluorescent markers, thereby offering a more optimal imaging solution.

**Figure 9:**
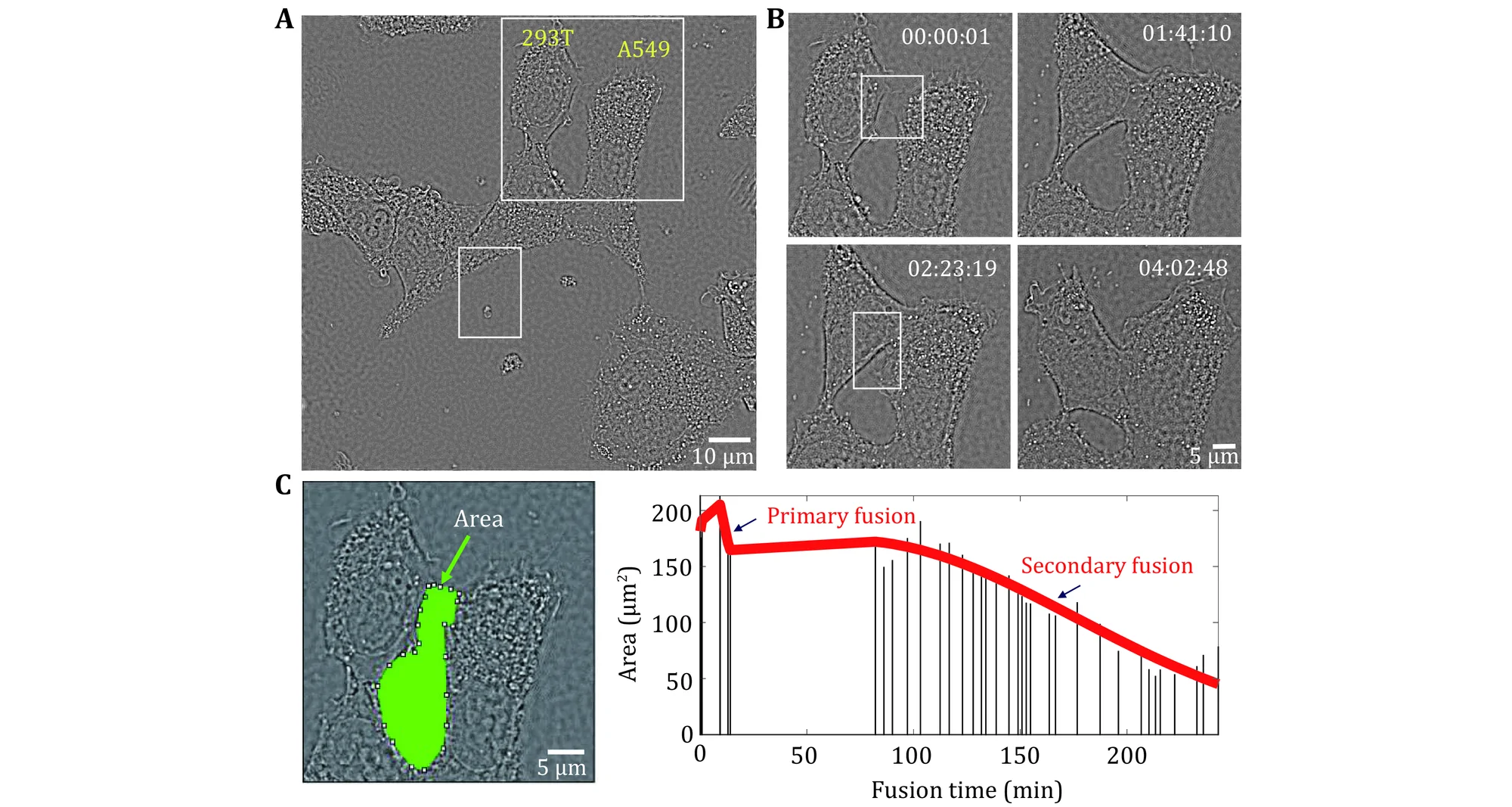
Label-free visualization of cell-cell fusion observed over a time period of 4 h. **A** Overall view of the fusion process between spike-GFP transfected HEK-293T cells and ACE2 expressed A549 cells. **B** Magnified regions enclosed by the upper white box in Panel A, illustrating the cell−cell fusion dynamic process at four-time points. **C** relationships between the fusion area cells fusion time

In Fig. 8, we show the fusion process between spike-GFP-transfected HEK-293T cells and ACE2-expressing A549 cells over a 4-h period. As the fusion progresses, the boundaries between the fused cells gradually become blurred or even vanish, and the fusion area continues to expand (Figs. 9A and 9B). We also plotted the relationship curve between the fused cell area and fusion time, analyzing the changes in fusion dynamics. Our results indicate that primary fusion occurs within the first 30 minutes, while secondary fusion happens 1−2 h later (Fig. 9C). With the label-free advantage of FPM technology, we were able to achieve long-term, continuous dynamic observation, enabling a more comprehensive study of the complex process of cell fusion.

## DISCUSSION AND OUTLOOK

In light of the recent developments in FPM, significant progress has been made in theoretical models, optimization algorithms, system construction, expansion, and applications. However, this does not imply that research in this direction has reached completion or saturation. There is still room for improvement, particularly in live cell imaging applications, with the following areas for further development:

(1) Enhancing imaging contrast and observing low refractive index organelles. To address the issue of insufficient contrast in imaging low refractive index organelles (such as the endoplasmic reticulum) with FPM, optimization will be carried out using multi-dimensional strategies. At the algorithmic level, improvements in phase recovery algorithms, combined with adaptive illumination control (such as introducing sparse constraints or deep learning priors), will enhance the ability to extract weak phase signals. At the hardware level, the integration of high dynamic range image sensors and polarization-sensitive illumination technology will be explored, using multi-physical field information fusion to improve contrast resolution. Additionally, the development of virtual staining algorithms based on organelle-specific labeling will enable the visualization of structures like the endoplasmic reticulum without relying on chemical stains.

(2) Integration of live cell culture and dynamic environmental control systems. To facilitate long-term observation of live cells, FPM systems need to be integrated with modular microenvironment control devices. By designing multi-layer microfluidic chips and thermostatic sample stages, combined with CO_2_ concentration gradient feedback systems and nano-heating elements, a dynamic culture platform capable of temperature control (±0.1°C precision), gas regulation (O_2_/CO_2_ dual channels), and nutrient supply will be built. At the same time, low-light toxicity illumination strategies (such as pulsed LED light sources) will be developed, in conjunction with real-time phase tracking algorithms, to enable high-throughput capture of subcellular dynamics while maintaining cell viability. This will provide a new paradigm for studying cellular stress responses.

(3) Development of multi-modal fluorescence-fpm co-imaging technology. By optimizing the optical path and the design of time/frequency-division acquisition systems, a spatial registration mechanism between FPM and super-resolution fluorescence imaging will be established. Fluorescent labeling will be used to specifically locate target organelles, synchronously acquiring both their refractive index distribution and molecular composition information. Cross-modal data fusion algorithms (such as feature-cascade neural networks) will be used to perform structural-functional correlation analysis. Furthermore, the integration of Stimulated Raman Scattering (SRS) and FPM will be explored to overcome the phototoxicity limitations of fluorescence labeling, providing technical support for multi-scale analysis of cellular metabolic processes.

These technological breakthroughs will push FPM from static imaging to dynamic multi-modal analysis systems, providing high-dimensional observation tools for cutting-edge research on organelle interaction mechanisms, subcellular physiological processes, and other related areas. Additionally, they will open up new technical pathways for pathological diagnosis and drug screening.
